# The complete chloroplast genome of apple rootstock ‘M9’

**DOI:** 10.1080/23802359.2019.1624642

**Published:** 2019-07-10

**Authors:** Xueqing Zhao, Ming Yan, Yu Ding, Xuesen Chen, Zhaohe Yuan

**Affiliations:** a Co-Innovation Center for Sustainable Forestry in Southern China, Nanjing Forestry University, Nanjing, China;; b College of Forestry, Nanjing Forestry University, Nanjing, China;; c State Key Laboratory of Crop Biology, Shandong Agricultural University, Tai’an, China

**Keywords:** Apple, M9, complete chloroplast genome, phylogenetic analysis

## Abstract

The dwarf M9 (*Malus domestica* ‘M9’) rootstock is the most widely available *Malus* rootstock, here we report the complete chloroplast (cp) genome of ‘M9’ rootstock. The size of the complete cp genome was 159,926 bp, with the large-copy (LSC, 88,065 bp) regions, small single-copy (SSC, 19,157 bp) regions, and two inverted repeat regions (IRs, 26,352 bp each). It contained 110 genes, including 78 protein-coding genes, 28 transfer RNA genes (tRNA), and 4 ribosomal RNA genes (rRNA). A phylogenetic tree demonstrated that ‘M9’ rootstock was closely related to *M. hupehensis*, *M. baccata*, *M. prunifolia*, *M. micromalus*, and *M. tschonoskii*.

Dwarf and intensive planting of apple trees have become common in many of the world’s production areas. Dwarfing stocks are the key elements of controlling apple tree height and ‘M9’ is one of the best available dwarfing stocks in commercial use, and makes tremendous contributions to apple production of the world (Webster and Wertheim 2003). ‘M9’ and other M series rootstocks were first under the names Doucin and Paradise for a long time before they were renamed by the experiment station at East Malling in England. The re-classification of these rootstocks resulted in many unclear points about the phylogeny of ‘M9’ and other apple cultivars in genus *Malus*. Recent availability of the complete cp genomes provides valuable information for the maternal lineage within species. Here, we determined the complete cp genome of the epidemic apple rootstock ‘M9’. The genome was deposited in GenBank with the accession number MK434916.

The voucher specimen was preserved in Shandong Agricultural University, Shandong Province, China (118°46′43″E, 32°02′38″N). Total genomic DNA was extracted using the DNeasy Plant Mini Kit (Qiagen, Venlo, the Netherlands). Paired-end genomic library was constructed and sequenced on Illumina HiSeq 2500 platform (Illumina, San Diego, CA). The raw paired-end reads were filtered and trimmed by Fastp program (Chen et al. 2018). The complete cp genome was *de novo* assembled using GetOrganelle (Ankenbrand et al. 2018) based on the obtained high-quality paired-end reads. After genome annotation by the online program GeSeq (Tillich et al. 2017), the annotation results were inspected by Geneious 8.0.4 (Kearse et al. 2012) and manually adjusted where necessary.

The complete cp genome of apple ‘M9’ was 159,926 bp in size. It consisted of a pair of inverted IR regions (26,352 bp each) separated by an LSC (88,065 bp) and an SSC (19,157 bp) regions. The overall GC contents were 36.6%. A total of 110 unique genes were found, including 78 protein-coding genes, 28 tRNAs, and 4 rRNAs, in which the total genes number was different from that in other reported *Malus* cp genomes (Bao et al. 2016; Hu et al. 2018; Zhang et al. 2018, 2019; Yan et al. 2019). Moreover, six protein-coding genes (*rpl2*, *rpl23, ycf2*, *ndhB*, *rps7*, and *rps12*), seven tRNA genes (*trnI-CAU*, *trnL-CAA*, *trnV-GAC*, *trnI-GAU*, *trnA-UGC*, *trnN-GUU*, and *trnR-ACG*), and all rRNA genes (*rrn4.5*, *rrn 5*, *rrn16*, and *rrn 23*) were located at the IR regions. Nine of the protein-coding genes and five of the tRNA genes contained introns, 12 of which contained one intron, two genes (*ycf3* and *clpP*) with two introns.

The compete cp genome sequences of other *Malus*, *Pyrus* and *Prunus* species were used to construct the phylogenetic tree, and *Vitis viniferia* as an outgroup. Multiple sequences alignment was executed by MAFFT (Katoh and Toh 2010). The maximum likelihood (ML) phylogenetic tree was constructed by the IQ-TREE with the best-fit model identified using ModelFinder (Nguyen et al. 2015; Kalyaanamoorthy et al. 2017). Figure 1 showed that ‘M9’ rootstock was closely related to the congeners *M. hupehensis*, *M. baccata*, *M. prunifolia*, *M. micromalus*, and *M. tschonoskii*. The availability of the complete cp genomes will provide additional information for the reconstruction of a phylogenetic model for the *Malus* species.

**Figure 1.**
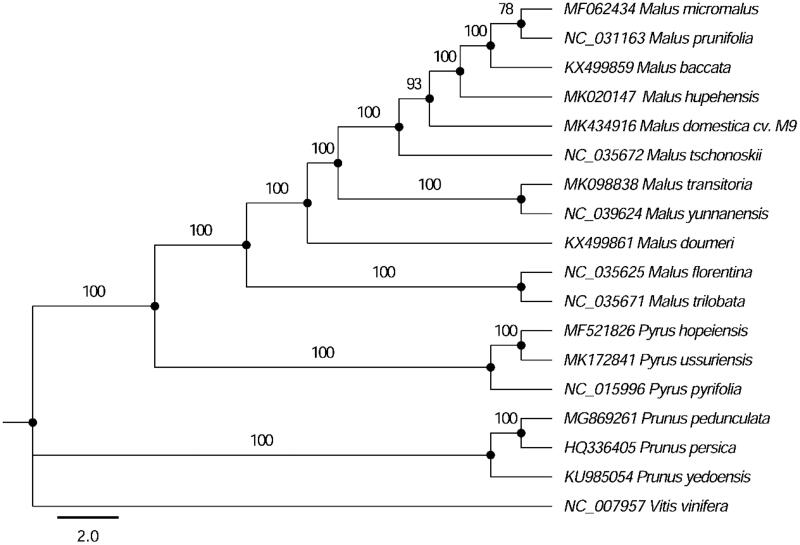
Phylogenetic tree of *Malus*, *Pyrus* and *Prunus* species with *Vitis vinifera* the outgroup using ML method. Numbers in the nodes are the bootstrap values from 1000 replicates.
